# Vitamin D receptor gene polymorphisms are associated with triceps skin fold thickness and body fat percentage but not with body mass index or waist circumference in Han Chinese

**DOI:** 10.1186/s12944-019-1027-2

**Published:** 2019-04-11

**Authors:** Fang Shen, Yan Wang, Hualei Sun, Dongdong Zhang, Fei Yu, Songcheng Yu, Han Han, Jun Wang, Yue Ba, Chongjian Wang, Wenjie Li, Xing Li

**Affiliations:** 1 Department of Nutrition and Food Hygiene, College of Public Health, Zhengzhou University, 100 Kexue Avenue, Henan, 450001 China; 2 Department of Occupational and Environmental Health Science, College of Public Health, Zhengzhou University, 100 Kexue Avenue, Henan, 450001 China; 3 Department of Epidemiological and Biostatistics, College of Public Health, Zhengzhou University, 100 Kexue Avenue, Henan, 450001 China

**Keywords:** *VDR* polymorphisms, Triceps skinfold thickness, Body fat percentage, Interaction, Adiposity

## Abstract

**Background:**

Evidence shows that low serum vitamin D concentrations account for an increased risk of obesity by inducing vitamin D receptor *(VDR)* hypofunction. Although the correlation between single nucleotide polymorphisms (SNPs) of *VDR* gene and obesity-related anthropometric measures (such as body mass index [BMI] and waist circumference[WC]) has already been tested, there are only few studies on the association between direct measures of body fat percentage (BFP) and triceps skinfold thickness and the SNPs of *VDR*. The aim of the present study was to evaluate the effect of *VDR* gene polymorphism on multiple obesity indexes in Han Chinese, including BMI, WC, BFP and triceps skinfold thickness.

**Methods:**

In this cross-sectional study, five hundred and seventeen healthy Chinese adults were enrolled in the trial. Four loci in *VDR* gene (rs2228570 [FokI], rs2189480, rs2239179 and rs7975232[ApaI]) were genotyped by TaqMan probe assays. Obesity indexes including BMI, WC, BFP and triceps skinfold thickness were used to evaluate the relationship to the *VDR* SNPs. Multiple logistic regression, linear regression and general multifactor dimensionality reduction (GMDR) were performed to analyze the correlation of *VDR* gene and obesity indexes.

**Results:**

None of the *VDR* SNPs were associated with BMI and WC, the C allele of FokI and the T allele of ApaI were associated with an increase in BFP (β = 0.069*,P* = 0.007; β = 0.087, *P* = 0.022 respectively); the G allele of rs2239179 and the T allele of ApaI were associated with an increase in triceps skin fold thickness (β = 0.074, *P* = 0.001; β = 0.122, *P* < 0.001 respectively). In regards to adiposity-related metabolic parameters, we found that the GT genotype of ApaI was associated with higher level of total cholesterol (TC) (*P* = 0.013) and Low-density lipoprotein cholesterol (LDL-C) (*P* = 0.001).

**Conclusions:**

Though we failed to prove that *VDR* SNPs were in correlation with BMI and WC, we did establish the association between *VDR* variants and BFP, as well as triceps skinfold thickness. Data obtained suggested that the *VDR* variants play an important role in regulating adipose tissue activity and adiposity among Han Chinese.

**Electronic supplementary material:**

The online version of this article (10.1186/s12944-019-1027-2) contains supplementary material, which is available to authorized users.

## Introduction

Worldwide obesity has nearly tripled since 1975. In 2016, more than 1.9 billion adults (aged 18 and older) were overweight, among which more than 30% were obese, triggering a pandemic [1]. Due to rapid changes in social politics, economy, culture, (For instance, experiencing a “nutrition transition”) obesity has become a serious issue for China in the past decades [2]. The accumulation of excess body fat contributes to higher morbidity and mortality rates of non-communicable diseases (NCDs), such as cardiovascular disease, chronic obstructive pulmonary disease, lung cancer, and diabetes [3, 4]. In order to assess the magnitude of the problem, the relevance between adiposity and NCDs are described using various anthropometric indicators such as body mass index (BMI), waist circumference (WC) and skinfold thickness. Previous studies have shown that high subcutaneous fat increases the risk of cardio-metabolic diseases independently [5, 6]. Meanwhile, individuals with a healthy body composition prefer to gain a healthier cardio-metabolic profile later on in life, and similar situations could be found in adult population with diabetes mellitus, hypertension, metabolic syndrome, and several of cancer [7–9].

Both vitamin D deficiency and insufficiency are common in China [10, 11]. Since negative correlation between serum 25(OH)D_3_ level and high adiposity has been well established [12–14], recent studies have focused on the physiological functions of vitamin D in adipose tissue [15–17]. It is well known that adipose tissue plays a pivotal role in energy balance and glucose homeostasis and serves as the main storage site for vitamin D [18]. Even human SAT (Subcutaneous adipose tissue) and VAT (Visceral adipose tissue) express *VDR* and enzymes involved in vitamin D metabolism [19]. Vitamin D is active in adipose tissues and act as a key regulator of gene expression, as well as signal transductions [20]. It is worth noting that vitamin D couples with its receptor (*VDR*) to exerts multiple biological functions and the binding of vitamin D to *VDR* plays an important role in regulating adipogenesis both in vivo and invitro [21, 22].

A body of studies have been conducted to investigate the association between VDR variants and different adiposity phenotypes [23–27], so far failed to reach a consensus. Furthermore, most of these studies were involved in anthropometric measurements (BMI and WC), but not obesity biomarkers of regional fat accumulation such as subcutaneous fat (skinfold thickness) or total fat accumulation such as body fat percentage[BFP]. As BMI and WC cannot distinguish fat from lean mass, explicit understanding of these two obesity biomarkers may provide important insights into the association between *VDR* variants and BMI or WC in different studies.

To our knowledge, the relationship between *VDR* variants and adiposity in Han Chinese are rarely investigated, let alone the regional and total body composition. Therefore, the possible relationships between single-nucleotide polymorphisms (SNPs) in *VDR* and multiple adiposity traits like BMI, WC, BFP and skin fold thickness are discussed in the study, Aiming to illustrate the biological significance of *VDR* polymorphisms on adiposity and may contribute to the identification of novel therapeutic strategies to prevent or treat adiposity and adiposity-related disorders among Han Chinese.

## Materials and methods

### Study population

Between June and July of 2013, a total of 1851 subjects were selected in the present cross-sectional study, which was conducted in Henan Province, a central area of China. Participants’ age ranged between 18 and 90 years. Exclusion criteria was chronic non-communicable diseases, acute diseases, taking vitamin D or calcium supplementation, and cholesterol-lowering medications. Finally, 517 (259 women and 258 men) subjects were eligible for analysis. The study was conducted in accordance with the guidelines set out in the Declaration of Helsinki and was approved by the Zhengzhou University Medical Ethics Committee. All the participants were given written informed consent before any collection of samples and data.

### Data collection

At enrollment, all participants received clinical examination and anthropometric measurements undertaken by trained observers. Additionally, a standard questionnaire was used to obtain sociodemographic data regarding age, dietary intake, physical activity, family history of obesity, and medication use. Height, WC and hip circumference were measured to the nearest 0.1 cm by using a metric scale; WC was measured at the midpoint between the last rib and the iliac crest. Triceps skinfold thickness was measured from the left side of the body to the nearest 0.1 mm, using a Holtain skinfold caliper (Holtain Ltd., Crymych, UK), at the flowing sites: halfway between the acromion process and the olecranon process. Weight and BFP were assessed by bioelectrical impedance analysis using the InBody 230 bioimpedance analyzer (Biospace CO Ltd., 518–10 Dogok2-dong, Gangnam-gu, Seoul, Korea). Weight was measured to the nearest 0.1 kg when the subjects were in their underwear and were not wearing shoes. BMI, waist-hip-ratio (WHR), waist-height-ratio (WHtR) were calculated as follows:

$$ \mathrm{BMI}=\mathrm{weight}\left(\mathrm{kg}\right)/\left({\mathrm{height}}^{\ast}\mathrm{height}\left(\mathrm{m}\right)\right) $$

$$ \mathrm{WHR}=\left(\mathrm{WC}\ \left(\mathrm{cm}\right)\right)/\left(\mathrm{Hip}\left(\mathrm{cm}\right)\right) $$

$$ \mathrm{WHtR}=\left(\mathrm{WC}\ \left(\mathrm{cm}\right)\right)/\left(\mathrm{height}\left(\mathrm{cm}\right)\right) $$

On the basis of Chinese BMI categories, subjects were divided into four groups: underweight (< 18.50 kg/m^2^), normal weight (18.50–23.99 kg/m^2^), overweight (24.00–27.99 kg/m^2^), and obesity (≥ 28.00 kg/m^2^) [28]. Abdominal obesity was defined as ≥90 cm for men and ≥ 80 cm for women [29].

Furthermore, after an overnight fast of at least 8 h, blood tests were used to determine the lipid profile (triglyceride [TG], total cholesterol [TC], high-density lipoprotein cholesterol [HDL-C]) by using an automatic biochemical analyzer (Shanghai Kehua Bio-engineering Co, Ltd. (KHB), Shanghai, China). Low-density lipoprotein cholesterol (LDL-C) was calculated based on the Friedewald equation.

### Selection and genotyping of SNPs

SNPs were screened from SNP haplotype map, NCBI database and a numerous review of literature. In addition, the selection of particular SNPs was based on the minor allele frequency (MAF) > 0.05, possible functional consequences and previous association with vitamin D level or indices of obesity or adiposity. Finally, we selected four SNPs, namely rs2228570 (FokI), rs2189480, rs2239179, rs7975232 (ApaI). Genomic DNA from peripheral blood was isolated by the standard procedures (DNA blood kit; Bioteke, Beijing, China). Genotyping involved use of an Applied Biosystems (7500 FAST Real-Time PCR system; Applied Biosystems, Foster City, USA).

### Statistical analysis

Continuous variables (normal distributed) were represented as mean ± standard deviation and were analyzed by student′s t-test, while quantitative data because of skewed distribution were illustrated as medians with corresponding quartile range and were analyzed by Mann-Whitney U-test. The Chi-squared test was used for categorical data. Departure from the Hardy-Weinberg equilibrium (HWE) was assessed in controls by using online software (http://shesisplus.bio-x.cn/SHEsis.html##).

Logistic regression for multivariate analyses was used to examine association between *VDR* variants and risk of overweight/obesity and abdominal obesity with adjustment for age, gender, family history of obesity, alcohol use, high-fat diet, vegetables consumption, and physical activity, assuming additive and dominant models of inheritance. Linear regression analysis was then used to examine the genetic association with the continuous outcomes (BFP, triceps skinfold thickness) with adjustment for age, gender, alcohol, high-fat diet, vegetable consumption, physical activity and the family history of obesity. One of the four possible models (additive, codominant, dominant and recessive) in the linear model, we selected the additive model because it generally reflects the additive contribution to risk for complex diseases. The relationship between the *VDR* polymorphism and adiposity-related metabolic parameters was analyzed by Analyses of Variance (ANOVA). Statistical values were performed with SPSS version 21.0 (IBM Corp., Chicago, IL, USA).

Generalized multifactor dimensionality reduction (GMDR) method permits adjustment for discrete and quantitative covariates and is applicable to both dichotomous and continuous phenotypes in various population-based study designs [30]. Analysis of the interaction on continuous phenotype (BFP and triceps skinfold thickness) between gene and gene, gene and environment was performed by GMDR (GMDR, version 0.7, University of Virginia, USA). The model with highest cross-validation consistency (CVC) and the maximum testing balanced accuracy was considered as a best model. Statistical tests were two-sided and *P*-values of < 0.05 were considered significantly.

## Results

### Basic characteristics

Clinical features and anthropometric measurements of the study subjects are shown in Table 1. Overweight and obesity group had higher levels of BMI, WC, WHR, WHtR, triceps skin fold thickness, BFP, LDL-C, TC, and TG than the normal weight group. Conversely, HDL-C was significantly lower in overweight and obesity group than normal weight group.

**Table 1:** Basic characteristics of the study participants

Parameter	Normal	Overweight/obesity	χ^2^/Z/t	*P*
	253	264		
Gender			0.855	0.353
Male	121(47.8%)	137(51.9%)		
Female	132(52.2%)	127(48.1%)		
Age			9.819	0.002
≤45	172(68.0%)	144(54.5%)		
> 45	81(32.0%)	120(45.5%)		
BMI (kg/m^2^)	21.55(19.81,22.80)	26.51(25.10,28.22)	−19.67	< 0.001
WC (cm)	75.71 ± 6.91	90.20 ± 8.12	−21.821	< 0.001
WHR	0.83 ± 0.06	0.91 ± 0.06	−14.53	< 0.001
WHtR	0.47 ± 0.05	0.56 ± 0.05	−22.52	< 0.001
Triceps skin fold thickness (mm)	15.48 ± 7.75	21.57 ± 8.29	−8.584	< 0.001
Body fat percentage (%)	19.05 ± 6.45	26.09 ± 6.49	−12.174	< 0.001
HDL-C (mmol/L)	1.42 ± 0.29	1.31 ± 08.27	3.656	< 0.001
LDL-C (mmol/L)	2.16 ± 0.73	2.39 ± 0.71	−3.500	0.001
TC (mmol/L)	4.04 ± 0.95	4.49 ± 1.04	−5.170	< 0.001
TG (mmol/L)	0.78(0.58,1.19)	1.33(0.84,2.14)	−8.127	< 0.001

Data are given as the mean ± SD, as n (%) or median (quartile range). BMI, body mass index; WC, waist circumference; WHR, waist-hip-ratio; WHtR, waist-height-ratio; HDL-C, high-density lipoprotein cholesterol; LDL-C, low-density lipoprotein cholesterol; TC, total cholesterol; TG, triglyceride

Data are given as the mean ± SD, as n (%) or median (quartile range). BMI, body mass index; WC, waist circumference; WHR, waist-hip-ratio; WHtR, waist-height-ratio; HDL-C, high-density lipoprotein cholesterol; LDL-C, low-density lipoprotein cholesterol; TC, total cholesterol; TG, triglyceride.

### Association analysis of overweight/obesity and abdominal obesity and the distribution of the *VDR* SNPs

**Fig. 1:**
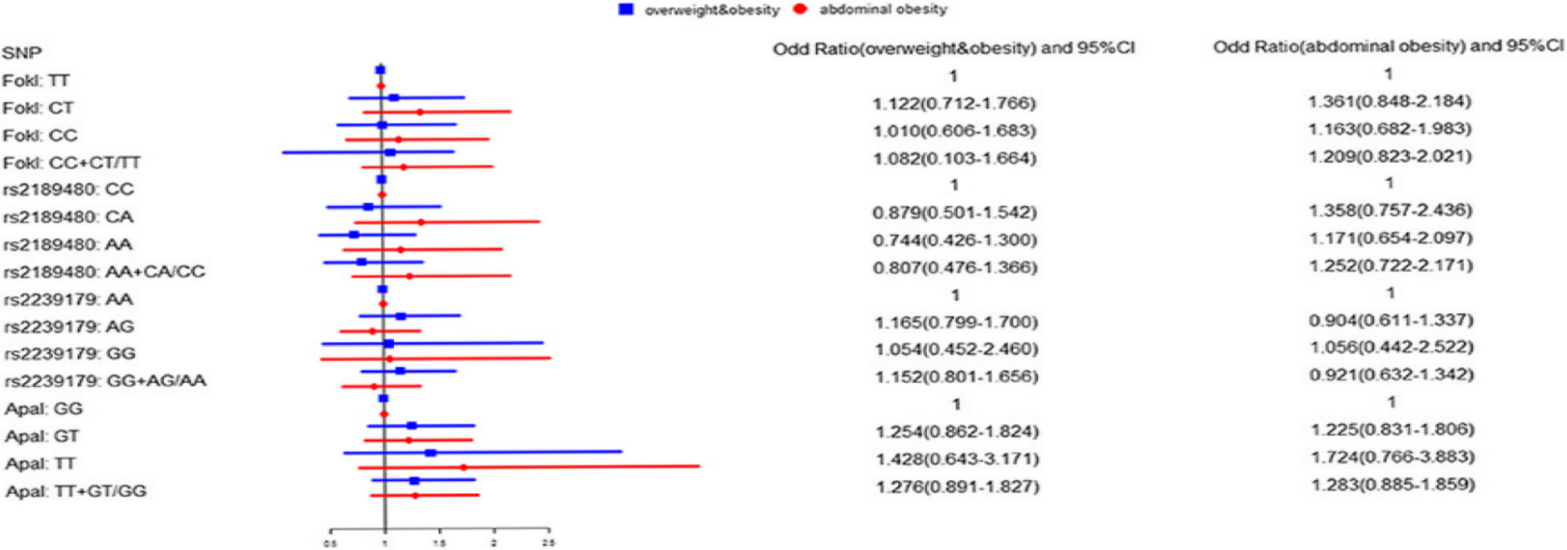
Forest plot of association between VDR polymorphisms and overweight/obesity and abdominal obesity. Logistic regression analyses were adjusted for age, gender, alcohol, high-fat diet, vegetable consumption, physical activity and the family history of obesity.

The allele and genotype distribution of FokI, rs2189480, rs2239179, and ApaI are given in Additional file 1: Table S1 and S2. The distribution of *VDR* polymorphisms in the control group obeyed HWE (*P* > 0.05). There were no significant differences in the frequency of genotypes at selected *VDR* polymorphisms. The covariate-adjusted associations of *VDR* SNPs with overweight/obesity and abdominal obesity are presented in Fig. 1 Under additive model and dominant models, none of the SNPs showed significant association with BMI or WC in the studied population.

### Linear regression-derived association of selected tagging *VDR* SNPs with BFP and triceps skinfold thickness in Han Chinese

The association of selected *VDR* SNPs with BFP and triceps skinfold thickness are presented in Table 2. Two SNPs (FokI and ApaI, rs2239179 and ApaI respectively) were significantly associated with BFP and triceps skinfold thickness. Assuming an additive genetic model on inheritance, for FokI (β ± SE = 0.069 ± 0.031, *P* = 0.007), we observed that each additional rare allele resulted in a 6.9% increase in BFP, and for ApaI (β ± SE = 0.087 ± 0.039, *P* = 0.022), each additional rare allele resulted in a 8.7% increase in BFP; simultaneously, for rs2239179 (β ± SE = 0.074 ± 0.028, *P* = 0.001), each additional rare allele resulted in a ~ 0.074-mm increase in triceps skinfold thickness, and for ApaI (β ± SE = 0.122 ± 0.034, *P* < 0.001), each additional rare allele resulted in a ~ 0.122-mm increase in triceps skinfold thickness.

**Table 2:** Selected tagging VDR SNPs significantly associated with body fat percentage and triceps skin fold thickness

Body fat percentage (%)			Triceps skinfold thickness (mm)	
SNPs	β ± SE	*P*	β ± SE	*P*
FokI	0.069 ± 0.031	0.007	0.051 ± 0.030	0.053
rs2189480	0.041 ± 0.029	0.148	0.049 ± 0.029	0.065
rs2239179	−0.030 ± 0.052	0.560	0.074 ± 0.028	0.001
ApaI	0.087 ± 0.039	0.022	0.122 ± 0.034	< 0.001

Results are presented as β ± SE, β coefficients represent the mean difference in body fat percentage and triceps skinfold thickness for each additional rare allele of the corresponding SNP. Linear regression analyses were adjusted for age, gender, alcohol, high-fat diet, vegetable consumption, physical activity and the family history of obesity

Results are presented as β ± SE, β coefficients represent the mean difference in body fat percentage and triceps skinfold thickness for each additional rare allele of the corresponding SNP. Linear regression analyses were adjusted for age, gender, alcohol, high-fat diet, vegetable consumption, physical activity and the family history of obesity.

### Higher BFP and triceps skinfold thickness susceptibility is due to the SNPs in *VDR* gene

Analysis of inter-gene (4SNPs) interaction obtained from GMDR was summarized in Table 3. The interaction model composed of FokI, rs2239179 and ApaI on the high BFP susceptibility was the best model (*P* < 0.001). Overall, the cross-validation consistency of this three-locus model was 10/10, and the testing accuracy was 57.5%. Also, we found the best interaction combination involving these four SNPs on the risk of higher skinfold thickness. Overall, the cross-validation consistency of this four-locus model was 10/10, and the testing accuracy was 54.1%. However, we did not find any significant interaction between the four SNPs and environmental factors (alcohol, high-fat dietary, exercise and family history of obesity) on these two phenotypes.

**Table 3:** GMDR models of gene-gene interactions on body fat percentage and triceps skin fold thickness

Models	TBA1	TBA2	CVC	*P*_*sign*_
Gene-gene interaction for body fat percentage^a^				
ApaI	0.569	0.511	6/10	0.172
FokI, ApaI	0.602	0.524	6/10	0.055
FokI, ApaI, rs2239179	0.632	0.575	10/10	0.010
FokI, ApaI, rs2239179, rs2189480	0.657	0.553	10/10	0.055
Gene-gene interaction for triceps skin fold thickness^a^				
ApaI	0.593	0.595	10/10	0.001
FokI, ApaI	0.607	0.525	5/10	0.055
FokI, ApaI, rs2189480	0.643	0.578	6/10	0.001
FokI, ApaI rs2239179, rs2189480	0.655	0.541	10/10	0.001

*GMDR* Generalized multifactor dimensionality reduction, *TBA1* Training balanced accuracy, *TBA2* Testing balanced accuracy, *CVC* Cross-validation consistency. *P*_*sign*_ *= P* value from sign test. ^a^ Adjusted for age, sex, alcohol, high-fat diet and the family history of obesity

### Adiposity-related metabolic parameters according to genotypes of *VDR* polymorphism

The effects of the genotype distributions of the four SNPs on metabolic parameters were presented in Table 4. The results revealed that GT genotype of ApaI was associated with higher TC (*P* = 0.013) and LDL-C (*P* = 0.001).

**Table 4:** Association of *VDR* polymorphisms with metabolic parameters

SNP	TG	TC	HDL-C	LDL-C
FokI	*P* = 0.924	*P* = 0.809	*P* = 0.611	*P* = 0.410
TT	1.385 ± 1.091	4.218 ± 0.983	1.348 ± 1.091	2.199 ± 0.695
CT	1.400 ± 1.310	4.293 ± 1.049	1.357 ± 0.283	2.312 ± 0.732
CC	1.445 ± 1.379	4.272 ± 1.012	1.382 ± 0.295	2.277 ± 0.751
rs2189480	*P* = 0.778	*P* = 0.948	*P* = 0.992	*P* = 0.231
CC	1.034 ± 1.075	4.290 ± 1.027	1.364 ± 0.296	2.378 ± 0.731
CA	1.425 ± 1.411	4.254 ± 1.027	1.360 ± 0.297	2.218 ± 0.665
AA	1.425 ± 1.220	4.282 ± 1.024	1.363 ± 0.272	2.306 ± 0.782
rs2239179	*P* = 0.093	*P* = 0.205	*P* = 0.611	*P* = 0.877
AA	1.357 ± 1.138	4.241 ± 1.019	1.353 ± 0.277	2.279 ± 0.734
AG	1.552 ± 1.551	4.361 ± 1.034	1.377 ± 0.292	2.290 ± 0.725
GG	1.015 ± 0.608	4.005 ± 0.976	1.399 ± 0.353	2.204 ± 0.733
ApaI	*P* = 0.933	*P* = 0.013	*P* = 0.961	*P* = 0.001
GG	1.426 ± 1.414	4.195 ± 0.998	1.363 ± 0.277	2.199 ± 0.670
GT	1.389 ± 0.992	4.437 ± 1.076^a^	1.363 ± 0.309	2.432 ± 0.822*
TT	1.358 ± 1.550	3.989 ± 0.783	1.347 ± 0.220	2.088 ± 0.461

Data are represented the mean ± SD. ^a^The second genotype is significantly different from the first genotype

## Discussion

In the present study, we investigated the effect of the external environment and inherent variations of *VDR* on obesity-related traits in humans. We found no significant link between *VDR* SNPs and anthropometric measures (BMI and WC), but positive associations of *VDR* SNPs with BFP and triceps skinfold thickness, FokI and ApaI for BFP, as well as rs2239179 and ApaI for triceps skinfold thickness. Furthermore, we identified significant gene-gene interactions with susceptibility to adiposity, three-locus model for BFP and four-locus model for triceps skinfold thickness. In addition, we found that *VDR* variants were related to adiposity-related metabolic complications. Hence, our study provides evidence that polymorphisms in *VDR* gene might play a role in regulating adipose tissue activity and susceptibility to adiposity among Han Chinese.

*VDR* is expressed in various types of adipose tissues such as 3 T3-L1 adipocyte, human preadipocytes, differentiated adipocyte, human SAT and VAT [31–33]. Adipose tissue has multiple functions of lipids synthesizing, fatty acids transporting and adipokine secretions [15]. It is reported that vitamin D treatment blocked adipogenesis in *VDR*^+/+^ cells but failed to do so in VDR^−/−^, indicating that *VDR* might be required for adipogenesis [34]. Also, previous studies have shown that *VDR* knockout mice shared lean phenotype and were resistance to diet-induced obesity, supporting the idea that 1α,25(OH)_2_-D_3_/*VDR* system modulates appetite and energy homeostasis [35, 36].

The association between *VDR* genetic variants and anthropometric measures (BMI and WC) have been previously investigated mainly on British, Polish, American, Spanish and Saudi Arabian population. However, the results remained inconclusive [23, 24, 26, 37]. It is noted that a study involving 5224 participants in the 1958 British birth cohort did not find any link between *VDR* SNPs and BMI and WC [23]. On the contrary, homozygous rare genotype was related to an increase in larger WC compared to common homozygous genotype in a sample of 1773 healthy American females [38]. In our study, there was no significant association between *VDR* SNPs and BMI and WC in Han Chinese. Such differences may be attributed to different ethnic backgrounds [39]. Alternatively, different disease-causing alleles predominate in different study populations or variation exists in the degree of linkage disequilibrium between the markers and the disease alleles [40].

From the physiological point of view, it is not the degree of excess weight (as is measured by, for example BMI and WC), but the degree of body fatness acted as the risk factor. Evidence has been reported that skinfolds maybe more sensitive than BMI in detecting adiposity [41], as skinfolds are more directly associated than BMI with subcutaneous fat. Moreover, BFP had significantly stronger associations with obesity-related biomarkers than BMI did [42]. Previous study proved that *VDR* heterozygous mice showed significantly less fat accumulation than wild-type mice [43], so we hypothesized that *VDR* SNPs may affect directly on body compositions (such as skinfold thickness and BFP) compared to indirect parameters (such as BMI and WC), which was verified by our results. We observed that the T allele of FokI and the T allele of ApaI were associated with an increase in BFP; the G allele of rs2239179 and the T allele of ApaI were associated with an increase in triceps skin fold thickness. Several lines of evidence should be considered while assessing the role of *VDR* in adipogenesis. One possible explanation is that animal study suggest that 1,25(OH)2D3/*VDR* signaling exert suppressive effect on brown adipocyte differentiation, whereas brown adipose tissue is expressed in adult humans, functioning in non-shivering thermogenesis by uncoupling ATP synthesis from respiration, which plays an important role in energy expenditure [22]. Another underlying mechanism was that 1,25-dihydroxyvitamin D stimulates adipose leptin production in a *VDR*-dependent manner, and protected transgenic mice from body adipose accumulation [44].

ApaI, rs2189480 and rs2239179 are located in the 3′ untranslated region (UTR) or intron region and are unlikely to cause a disease. The FokI variants, located in the codon initiating translation that results in a smaller protein with increased capacity of 1,25-dihydroxyvitamin D binding [45], and accelerating adipogenesis in primary mouse and human preadipocytes, which was evidenced by increased expressions of adipogenic markers and lipid accumulation [46]. Therefore, analyses on VDR SNPs and their interrelations are being demanded as they may affect expression and activity of VDR. Our results suggested that the interactions between these four SNPs variants may lead to adiposity through linkage disequilibrium (LD), which may extend into 3’regulatory region (containing of the UTR); whereas polymorphisms in the 3’UTR region regulate gene expression by modulating messenger RNA stability and hence likely to affect the intracellular level of *VDR* [47]. Meanwhile the vitamin D signaling and adipogenesis are affected by *VDR* expression in a concentration-depending manner [21]. The relationship has established between SNPs in *VDR* gene and the body compositions so far, and the mechanism of which needs to be verified in future studies.

Emerging evidence indicates that variations in the *VDR* polymorphisms may contribute to dyslipidemia. In the present study, the ApaI homozygous rare genotype displayed a significant association with increased levels of TC and LDL-C. Also supported by Jia et al’ s conclusion as *VDR* SNPs are significantly correlated with risk of dyslipidemia and serum LDL levels in Chinese Han population [48]. In obese people, enhanced secretion of triglyceride-rich lipoprotein and impaired clearance of these lipoproteins increase the accumulation of visceral adiposity [49]. Furthermore, VDR mutational alleles carriers are commonly related to lower levels of serum 25(OH)D, higher lipid parameters and abnormal inflammatory biomarker in obese individuals [50]. The action of vitamin D is mediated through vitamin D receptor, a nuclear transcription-regulating factor that regulates de novo lipid synthesis, thereby contributing to the development of obesity [51]. Hence, *VDR* gene may influence the progression of adiposity activity via dyslipidemia.

There are several limitations to the present study including a lack of other genes that contribute to synthesis, transportation and degradation form of vitamin D such as CYP2R1, GC, CYP27B1 and CYP24A1. Another potential limitation is that associations with intermediate parameters such as adipokine secretion including leptin and adiponectin could have strengthened our findings; however, we did not have such data. Nevertheless, our study do have some strengths. First of all, our participants were all Han Chinese, eliminating population admixture as a potential drawback. Then, we excluded participants who had taken vitamin D/calcium supplements, avoiding external environmental interference. Last of all, one of the main strengths of our study was that we measured skinfold thickness and BFP which directly reflect adiposity accumulation. Thus we were able to study the association between *VDR* polymorphisms and the most active form of adiposity, which is not focused on by previous studies. The findings of this study may provide a new insight for association of *VDR* polymorphism and adiposity.

## Conclusions

In summary, our study suggests that *VDR* variants are associated with susceptibility to adiposity in Han Chinese. The genetic factors that contribute to adiposity are certainly more complex than to be explained entirely by variations in a single gene. We acknowledge that the results are limited by ethnic specificity and relatively small sample size, so these results need to be replicated and confirmed in a large-scale study and more potential functional *VDR* polymorphisms should be detected.

## Additional file

Additional file 1: **Table S1.** Allele and genotype frequencies of *VDR* polymorphism and risk of overweight and obesity. **Table S2.** Allele and genotype frequencies of *VDR* polymorphism and risk of abdominal obesity (DOCX 20 kb)

## Abbreviations

BFP: Body fat percentage
BM: Body mass index
CVC: Cross-validation consistency
GMDR: General multifactor dimensionality reduction
HDL-C: High-density lipoprotein cholesterol
HWE: Hardy-Weinberg equilibrium
LD: Linkage disequilibrium
LDL-C: Low-density lipoprotein cholesterol
NCDs: Non-communicable diseases
SAT: Subcutaneous adipose tissue
SNPs: Single nucleotide polymorphisms
TBA1: Training balanced accuracy
TBA2: Testing balanced accuracy
TC: Total cholesterol
TG: Triglyceride
UTR: Untranslated region
VAT: Visceral adipose tissue
*VDR*: Vitamin D receptor
WC: Waist circumference
WHR: Waist-hip-ratio
WHtR: Waist-height-ratio
