# Supplementary material for: Vitamin D receptor gene polymorphisms are associated with triceps skin fold thickness and body fat percentage but not with body mass index or waist circumference in Han Chinese
Source: Lipids Health Dis. 2019 Apr 11;18:97. doi: 10.1186/s12944-019-1027-2 (PMC6460735; doi:10.1186/s12944-019-1027-2)
Supplement: Supplementary file 1 — Table S1. Allele and genotype frequencies of VDR polymorphism and risk of overweight and obesity. Table S2. Allele and genotype frequencies of VDR polymorphism and risk of abdominal obesity (DOCX 20 kb) [file 12944_2019_1027_MOESM1_ESM.docx]

Table S1 Allele and genotype frequencies of *VDR* polymorphism and risk of overweight and obesity.

| SNP | Normal* | Overweight/obesity* | χ^2^ | *P* | HWE |
| --- | --- | --- | --- | --- | --- |
| FokI |  |  | 0.584 | 0.747 | 0.999 |
| TT | 57(22.53) | 54(20.45) |  |  |  |
| CT | 126(49.8) | 140(53.03) |  |  |  |
| CC | 70(27.67) | 70(26.52) |  |  |  |
| rs2189480 |  |  | 1.095 | 0.578 | 0.502 |
| CC | 31(12.25) | 38(14.39) |  |  |  |
| CA | 103(40.7) | 113(42.80) |  |  |  |
| AA | 119(47.0) | 113(42.80) |  |  |  |
| rs2239179 |  |  | 0.764 | 0.682 | 0.858 |
| AA | 158(62.45) | 157(59.47) |  |  |  |
| AG | 82(32.41) | 95(35.98) |  |  |  |
| GG | 13(5.14) | 12(4.55) |  |  |  |
| ApaI |  |  | 2.215 | 0.330 | 0.995 |
| GG | 156(61.66) | 146(55.30) |  |  |  |
| GT | 85(33.60) | 102(38.64) |  |  |  |
| TT | 12(4.74) | 16(6.06) |  |  |  |

^*^Data are given as n (%)

SNP, single nucleotide polymorphism; HWE, Hardy-Weinberg equilibrium.

Table S2 Allele and genotype frequencies of *VDR* polymorphism and risk of abdominal obesity.

| SNP | Normal* | Abdominal* | χ^2^ | *P* | HWE |
| --- | --- | --- | --- | --- | --- |
| FokI |  |  | 1.383 | 0.501 | 0.981 |
| TT | 67(23.0) | 44(19.5) |  |  |  |
| CT | 143(49.1) | 123(54.4) |  |  |  |
| CC | 81(27.8) | 59(26.1) |  |  |  |
| rs2189480 |  |  | 1.702 | 0.427 | 0.113 |
| CC | 42(14.4) | 27(11.9) |  |  |  |
| CA | 116(39.9) | 100(44.2) |  |  |  |
| AA | 133(45.7) | 99(43.8) |  |  |  |
| rs2239179 |  |  | 0.002 | 0.999 | 0.999 |
| AA | 177(60.8) | 138(61.1) |  |  |  |
| AG | 101(34.7) | 76(33.6) |  |  |  |
| GG | 13(4.5) | 12(5.3) |  |  |  |
| ApaI |  |  | 2.795 | 0.247 | 0.997 |
| GG | 179(61.5) | 123(54.4) |  |  |  |
| GT | 99(34.0) | 88(38.9) |  |  |  |
| TT | 13(4.5) | 15(6.6) |  |  |  |

^*^Data are given as n (%)

SNP, single nucleotide polymorphism; HWE, Hardy-Weinberg equilibrium.
